# Identification and validation of a nomogram predicting cancer-specific survival for elderly patients with adult fibrosarcoma: a multicenter retrospective study

**DOI:** 10.3389/fonc.2023.1187942

**Published:** 2023-07-12

**Authors:** Zhangheng Huang, Zhen Zhao, Yuheng Liu, Zhigang Zhou, Weifei Zhang, Qingquan Kong

**Affiliations:** ^1^ Department of Orthopedic Surgery, West China Hospital, Sichuan University, Chengdu, Sichuan, China; ^2^ Department of Orthopaedics, Jiujiang First People’s Hospital, Jiujiang, Jiangxi, China

**Keywords:** adult fibrosarcoma, seniors, nomogram, retrospective study, prediction model

## Abstract

**Background:**

Due to the low incidence of adult fibrosarcoma (AFS), it is difficult for clinicians to assess cancer-specific survival (CSS) in elderly patients based on this study. The study aimed to develop nomograms capable of accurately predicting 3-, 5-, and 8-year CSS in patients over 40 years of age with AFS.

**Methods:**

Data were collected from The Surveillance, Epidemiology, and End Results (SEER) registry. 586 patients were included in this study. Univariate as well as multivariate Cox regression analyses were applied to identify independent risk factors. A nomogram was constructed and validated to predict the 3-, 5-, and 8-year CSS of patients.

**Results:**

Five variables including age, sex, stage, grade, and chemotherapy status were considered independent risk factors and were used to construct the nomogram. The nomogram was well validated. The C-indexes of the training cohort and the validation cohort are 0.766 and 0.780, respectively. In addition, the area under the curves for 3-, 5- and 8-year CSS are 0.824, 0.846 and 0.840 in the training cohort, 0.835, 0.806 and 0.829 in the validation cohort. Calibration curves were also plotted to show that predicted endings have a well fit for the true endings. Finally, decision curve analysis demonstrates that the nomogram can bring a high benefit to patients.

**Conclusion:**

We successfully constructed a highly accurate nomogram to predict the CSS of AFS patients at 3-, 5-, and 8 years. The nomogram can greatly help clinicians and patients with AFS.

## Introduction

Adult fibrosarcoma (AFS) is a rare tumor consisting of malignant spindle-shaped fibroblasts. (1) It is classified by the World Health Organization as a fibroblastic and myofibroblastic tumor and is defined as a diagnosis of exclusion (2–4). What needs to be distinguished from infantile fibrosarcoma (IFS) is that IFS occurs primarily in children under 24 months of age (4–6). AFS occurs mostly in the median age of 50 years and is extremely rare in the adolescent population (3).

AFS was once incorrectly considered to be the most common soft tissue sarcoma. 66% of all sarcoma patients diagnosed at the Mayo Clinic between 1910 and 1930 were considered to have AFS (3). However, with the development of experimental diagnostic techniques, the incidence of AFS has declined rapidly. According to current reports, AFS accounts for less than 3.6% of all soft tissue sarcomas (3, 7, 8). Due to the extremely low incidence of AFS, it is easily overlooked by clinicians.

Relying only on the TNM staging system and AJCC staging system can’t meet the needs of clinicians to accurately predict patient prognosis. The nomogram is a visual model that integrates risk factors and assesses individual survival (9). It has been used in the prognosis prediction of a variety of cancers (10–12). Therefore, the study aimed to screen for risk factors that can influence cancer-specific survival (CSS) in elderly people over 40 years old with AFS and to develop a nomogram that can accurately predict the incidence of CSS at 3-, 5-, and 8-year.

## Methods

### Collection and selection of patients

The Surveillance, Epidemiology, and End Results (SEER) registry program collects patients’ information from 18 cancer registries, covering about 28% of the US population. All of the patient’s information in this study was obtained in the SEER database *via* SEER∗Stat Software Version 8.3.6.

**Figure 1:**
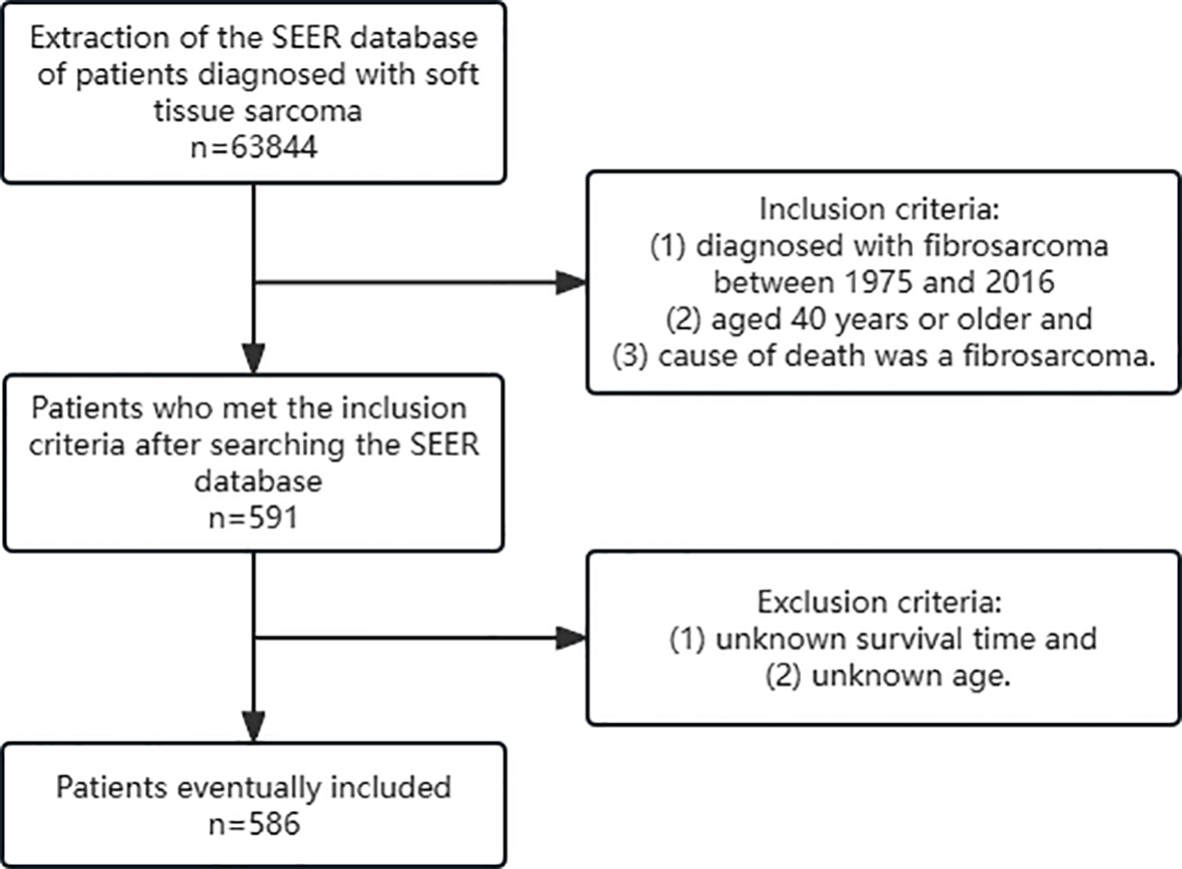
Screening process.

Inclusion criteria were as follows: (1) diagnosed with fibrosarcoma between 1975 and 2016 (2) aged 40 years or older and (3) cause of death was a fibrosarcoma. Exclusion criteria were as follows: (1) unknown survival time and (2) unknown age. At last, 586 patients were included in this retrospective study (**Figure 1**).

### Characteristics of patients

We collected clinicopathological information on patients including patients’ id, survival time, survival status, sex, age, race, tumor primary site, stage, grade, radiotherapy status, and chemotherapy status. The optimal cutoff point for age grouping is selected by the X-tile software (40-57, 58-69,>69). The race was categorized as black, white, and other. The pathological grade into three categories including grades I&II, grades III& IV, and unknown. According to “SEER historic stage A (1973-2015)”, the tumor stage was divided into localized, regional, distant and unknown.

### Statistical analysis

Patients were divided into a training cohort and a validation cohort in a ratio of 7:3 by R software (version 4.2.1). IBM SPSS Statistics 26.0 software was used to screen risk factors by univariate analysis cox proportional-hazards model (Cox). The endpoint was defined as CSS. Then these risk factors were included in a multivariate Cox analysis to exclude confounding effects between variables. The obtained independent risk factors were used to construct the nomogram. The C-index was calculated to estimate the prediction accuracy of the nomogram. The receiver operating characteristic (ROC) curve and the area under the curve (AUC) was used to evaluate the predictive ability of the nomogram. AUC is a valid method to summarize the overall diagnostic accuracy of a test. It has a range of values from 0 to 1, where a value of 0 indicates a completely inaccurate test and a value of 1 indicates a completely accurate test. A value of AUC between 0.7 and 0.8 is considered acceptable in the accepted statistical significance assessment, and the closer to 1 the better (1). Calibration curves were used to demonstrate the degree of fitness of predicted endings to true endings. In addition, decision curve analysis (DCA) was also applied to evaluate the actual benefit of this nomogram bringing to patients. Finally, we calculated the nomogram score of the patients. The X-tile software was used to calculate the best cutoff point for the nomogram score. We classified the patients into three risk subgroups: high, middle, and low, based on the cut-off points obtained. Kaplan-Meier curves were plotted to observe the prognosis of the three risk subgroups. The significant prognostic gap between different subgroups proves that this nomogram has a well prognostic prediction. All plots above were plotted using R software (version 4.2.1) and were considered statistically significant when the p-value was less than 0.05.

## Results

### Patients’ demographic and pathological characteristics

A total of 586 patients were included in this study according to the inclusion and exclusion criteria. Patients were randomized in a 7:3 ratio into a training cohort (n=412) and a validation cohort (n=174). Of these patients, 50.3% were between 40-57 years of age, 23.5% were between 58-69 years of age, and 26.1% were over 69 years of age. More details are presented in **Table 1**.

**Table 1:** Demographic and clinical characteristics of elderly patients with AFS.

Variables	Total cohort		Training cohort		Validation cohort	
	N=586		N=412		N=174	
	n	%	n	%	n	%
Age						
40-57	295	50.3	207	50.2	88	50.6
58-69	138	23.5	99	24.0	39	22.4
>69	153	26.1	106	25.7	47	27.0
Race						
Black	84	14.3	58	14.1	26	14.9
White	458	78.2	317	76.9	141	81.0
Other	44	7.5	37	9.0	7	4.0
Sex						
Male	314	53.6	222	53.9	92	52.9
Female	272	46.4	190	46.1	82	47.1
Marital status						
No	233	39.8	158	38.3	75	43.1
Yes	353	60.2	254	61.7	99	56.9
Primary site						
Bone and joints	62	10.6	45	10.9	17	9.8
Soft tissue	524	89.4	367	89.1	157	90.2
Grade						
I&II	220	37.5	165	40.0	55	31.6
III&IV	188	32.1	124	30.1	64	36.8
Unknown	178	30.4	123	30.0	55	31.6
Stage						
Localized	292	49.8	206	50.0	86	49.4
Regional	145	24.7	100	24.3	45	25.9
Distant	75	12.8	53	12.9	22	12.6
Unstaged/unknown	74	12.6	53	12.9	21	12.1
Radiotherapy						
No	388	66.2	280	68.0	108	62.1
Yes	198	33.8	132	32.0	66	37.9
Chemotherapy						
No	482	82.3	338	82.0	144	82.8
Yes	104	17.7	74	18.0	30	17.2

### Prognostic risk factors for CSS

After univariate Cox regression analysis, age, sex, tumor primary site, stage, grade, and chemotherapy status were shown to be prognostic risk factors for CSS. The above risk factors were included in the multivariate Cox regression analysis. After excluding the effect of confounders among variables, age (58-69, HR= 0.241, 95%CI= 0.171-0.341, P< 0.001; ≥69, HR= 0.482, 95%CI= 0.333-0.697, P< 0.001), sex (female, HR= 1.486, 95%CI= 1.117-1.977, P= 0.007), stage (regional, HR= 0.453, 95%CI= 0.286-0.718, P=0.001;unstaged/unknown, HR= 2.890, 95%CI= 1.726-4.837, P< 0.001), grade (III&IV, HR= 0.445, 95%CI= 0.310-0.640, P< 0.001), and chemotherapy status (yes, HR= 0.661, 95%CI= 0.463-0.945, P= 0.023)were screened out as independent CSS prognostic influences (**Table 2**), but tumor primary site is no longer significant.

**Table 2:** Analysis of univariate and multivariate Cox regression in elderly patients with AFS.

Characteristics	Univariate analysis		Multivariate analysis	
	HR (95% CI)	P value	HR (95% CI)	P value
Age				
40-57	Reference		Reference	
58-69	0.267 (0.192-0.371)	<0.001	0.241 (0.171-0.341)	<0.001
≥69	0.543 (0.382-0.771)	0.001	0.482 (0.333-0.697)	<0.001
Race				
Black	Reference			
White	0.902 (0.475-1.711)	0.752		
Other	1.191 (0.702-2.022)	0.516		
Sex				
Male	Reference		Reference	
Female	1.479 (1.117-1.959)	0.006	1.486 (1.117-1.977)	0.007
Marital status				
No	Reference			
Yes	1.018 (0.765-1.354)	0.903		
Primary site				
Bone and joints	Reference			
Soft tissue	1.763 (1.195-2.599)	0.004		
Grade				
I&II	Reference		Reference	
III&IV	0.357 (0.252-0.506)	<0.001	0.445 (0.310-0.640)	<0.001
Unknown	0.805 (0.582-1.112)	0.188	0.812 (0.578-1.140)	0.229
Stage				
Localized	Reference		Reference	
Regional	0.417 (0.269-0.649)	<0.001	0.453 (0.286-0.718)	0.001
Distant	0.730 (0.459-1.162)	0.184	0.710 (0.438-1.154)	0.167
Unstaged/unknown	2.701 (1.676-4.354)	<0.001	2.890 (1.726-4.837)	<0.001
Radiotherapy				
No	Reference			
Yes	1.167 (0.865-1.575)	0.312		
Chemotherapy				
No	Reference		Reference	
Yes	0.491 (0.358-0.673)	<0.001	0.661 (0.463-0.945)	0.023

### Construction and validation of a nomogram

**Figure 2:**
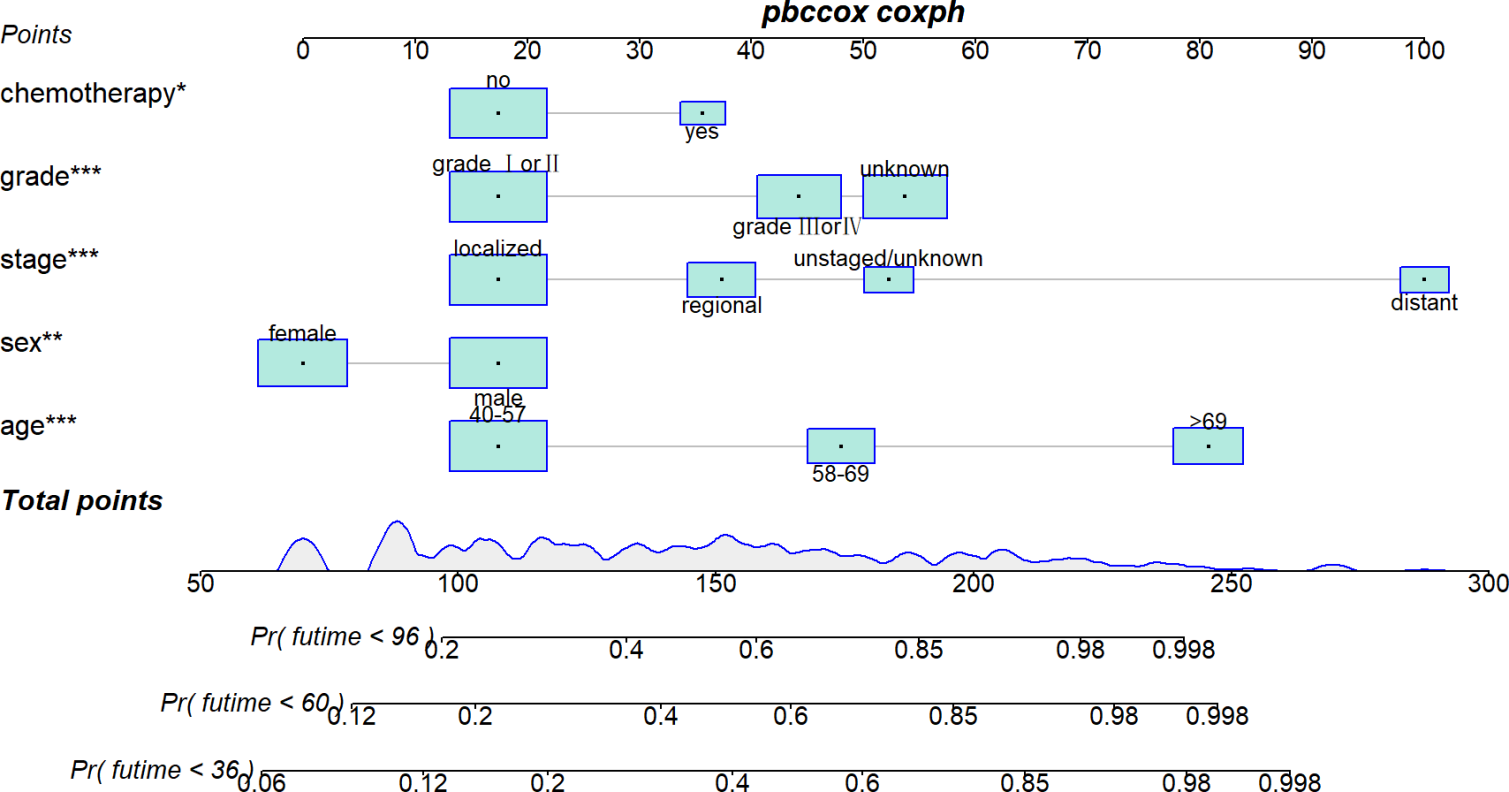
A nomogram for predicting the cancer-specific survival (CSS) in elderly patients with AFS.

**Figure 3:**
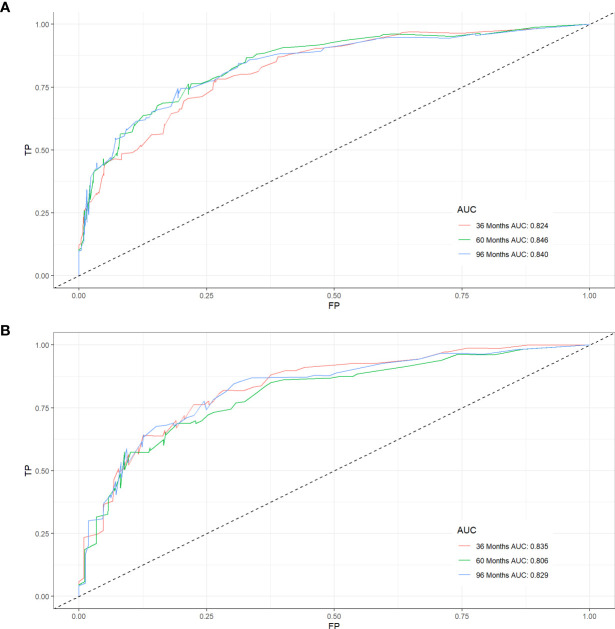
ROC curves for CSS prediction of elderly patients with AFS. **(A)** ROC curves of 3-, 5-, and 8-years in the training cohort, **(B)** ROC curves of 3-, 5-, and 8-years in the validation cohort.

**Figure 4:**
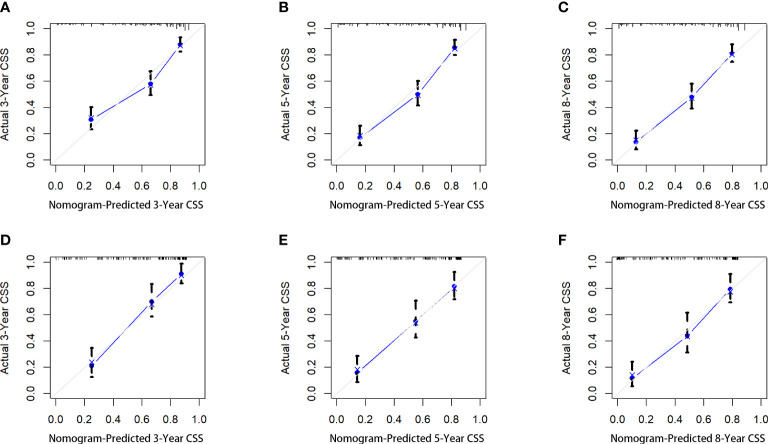
The calibration curves of the nomogram for predicting the 3-year, 5-years, and 8-years the CSS of the training cohort **(A–C)** and the validation cohort **(D–F)**.

**Figure 5:**
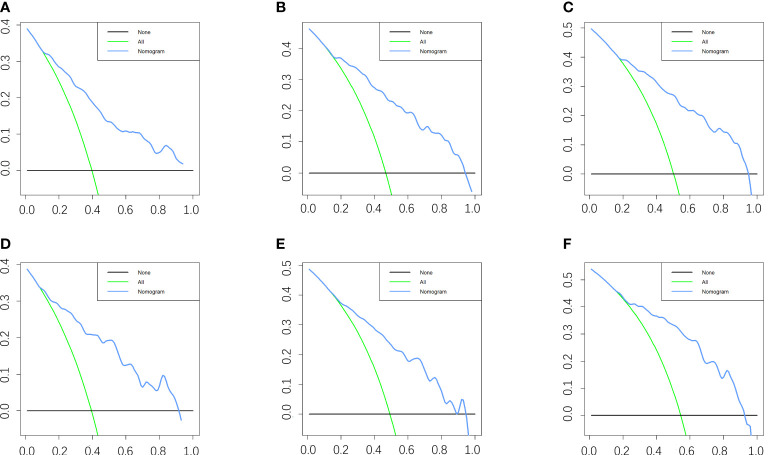
DCA of the nomogram for predicting the 3-, 5- and 8- years CSS in the training cohort **(A–C)** and the 3-, 5- and 8- years CSS in the validation cohort **(D–F)**.

Five variables were used to construct the nomogram, including age, sex, stage, grade, and chemotherapy status (**Figure 2**). After calculation, the C-index is 0.766 in the training cohort and 0.780 in the validation cohort. This indicates that the prediction results of the nomogram have high accuracy. The AUCs for 3-, 5- and 8-year CSS are 0.824, 0.846 and 0.840 in the training cohort, 0.835, 0.806 and 0.829 in the validation cohort (**Figure 3**). In addition, calibration curves show that predicted endings have a well fit for the true endings (**Figure 4**). Finally, DCA demonstrates that the nomogram can bring a benefit to the patient (**Figure 5**). It dedicates that the nomogram has a high clinical application value.

### Risk stratification

**Figure 6:**
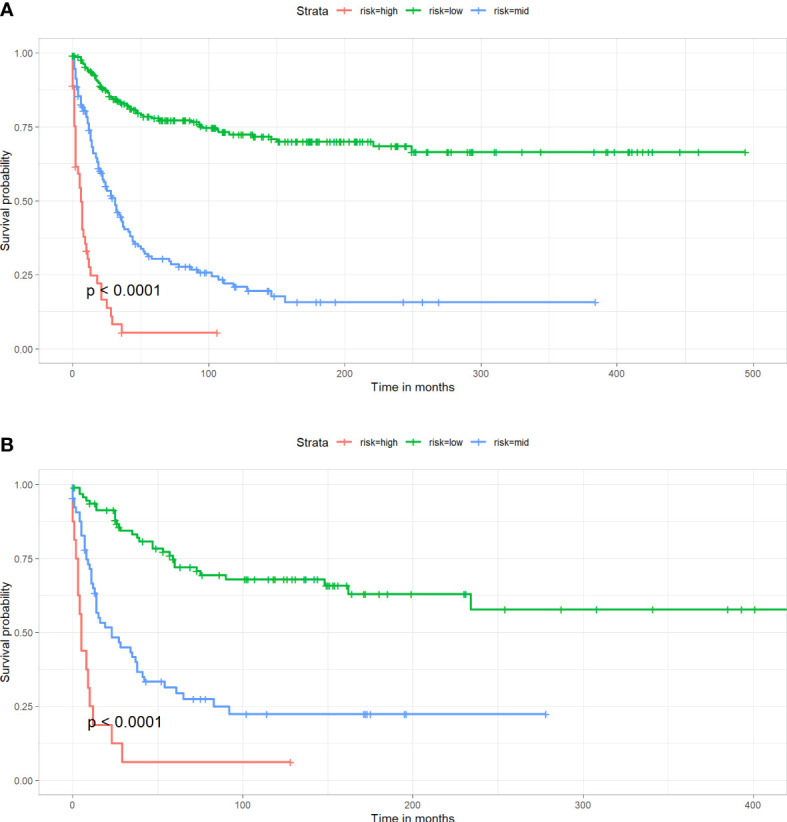
Kaplan-Meier survival curve for patients in different risk groups in both cohorts. **(A)** Training cohort, **(B)** Validation cohort.

By X-tile software, the best cutoff points were confirmed. Patients with nomogram scores below 144 were defined as low-risk patients, with nomogram scores above 207 were defined as high-risk patients, and the remaining patients were defined as mid-risk patients. As shown in **Figure 6**, the prognosis of CSS varies significantly among patients in different risk groups. This can help clinicians quickly obtain prognosis information of different patients.

## Discussion

Our study focused on cancer-specific survival, which means that the results will be more representative in the treatment and evaluation of AFS. We found that age, sex, stage, grade, and chemotherapy status were independent risk factors affecting CSS of elderly people over 40 years old with AFS. Age is thought to be associated with poor prognosis in many cancers (13–15).

In previous studies, age has been reported to be an onset predisposing factor and an important prognostic risk factor for AFS in related studies (16–18). This may be related to the accumulation of harmful substances and long-term exposure to risk factors. As seen in our nomogram (**Figure 2**), except for distant tumor metastasis, high age was the factor that allowed patients to obtain the highest nomogram score. This indicates that higher-age patients deserve more attention, which is the purpose of our study.

Many reports suggest that AFS has a higher incidence in the male population (19). However, this is not enough to prove that men are a risk factor for the prognosis of AFS patients. In a retrospective study by Xiang et al., they obtained the same conclusion as ours (7). Interestingly, in the report by Criscito et al. on dermatofibrosarcoma, men were found to be an important prognostic risk factor and it was noted that men were more likely to develop head and neck tumors (20). Since the data for the relevant retrospective studies were obtained from the SEER database, this may have led to bias. But it is also possible that new clinical findings, which need to be confirmed by more studies.

Similar to other sarcomas, multivariate Cox regression analysis showed that tumor stage and tumor grade were also independent risk factors in AFS (21–23). Distant metastases and high-grade of tumors indicate the widespread spread of tumor cells and multi-organ involvement. Bahrami et al. suggested that more than 80% of AFS patients had high-grade tumors (19). It indicates that without early diagnosis the patient’s survival expectancy will be significantly reduced. In addition, our study also found that not receiving chemotherapy was also a risk factor for CSS in patients over forty years of age with AFS. Although AFS has a low sensitivity to chemotherapy, undergoing surgery and chemotherapy is also the best treatment option for patients with AFS (4). Doxorubicin in combination with other chemotherapeutic agents is regarded as the most common drug applied to patients. While AFS has a low sensitivity to radiotherapy, we didn’t find an effect of radiotherapy on patients’ CSS, which is consistent with the findings of other scholars (20, 24). The use of radiotherapy for patients with AFS is still controversial, so it is not a standard treatment (25, 26).

We still need to acknowledge the limitations of our study. The low prevalence of AFS resulted in an insufficiently rich sample size, which may have led to some bias in our study. But we still have a better validation of the nomogram. This demonstrates that this nomogram has good clinical predictive power and can provide assistance to clinicians.

## Conclusion

Age, sex, stage, grade and chemotherapy status were shown to be risk factors affecting 3-, 5- and 8-year CSS in patients with AFS. The above five variables were used to construct the nomogram and were well validated. The development of the nomogram can provide a powerful aid to clinicians.

## Data availability statement

The original contributions presented in the study are included in the article/supplementary material. Further inquiries can be directed to the corresponding author.

## Ethics statement

Written informed consent was obtained from the individual(s), and minor(s)’ legal guardian/next of kin, for the publication of any potentially identifiable images or data included in this article.

## Author contributions

ZH and QK conceived and designed the study. ZH and ZZ performed the literature search. ZH and YL generated the figures and tables. ZH, ZGZ, and WZ analyzed the data. ZH wrote the manuscript and QK critically reviewed the manuscript. ZH and QK supervised the research. All authors contributed to the article and approved the submitted version.
